# Applications of RNA structure analysis to retroviral packaging and anti-retroviral therapeutic discovery

**DOI:** 10.1186/1742-4690-8-S2-O1

**Published:** 2011-10-03

**Authors:** Kevin M Weeks, Ben Berkhout, Julian W Bess, Siddhartha AK Datta, Cristina Gherge, Robert J Gorelick, Stefanie A Knoepfel, Christopher W Leonard, Tania Lombo, Justin T Low, Alan Rein, Olivier ter Brake, Joseph M Watts

**Affiliations:** 1 Department of Chemistry, University of North Carolina, Chapel Hill, NC 27599-3290, USA

## 

Our laboratory is blending new RNA chemistries, largely directed at the ribose 2'-hydroxyl group, with quantitative interpretation to create new kinds of chemical microscopes for accurate RNA structure analysis. This seminar will focus on two examples from retroviral biology. All retroviral RNAs contain a packaging element that directs the genome into nascent virus particles through a mechanism that has been unclear. Using SHAPE, we probed the structure of murine leukemia virus RNA inside virus particles and identified the binding site for the nucleocapsid domain of Gag. The RNA structural context is critical: High-affinity binding requires two specific UCUG-UR-UCUG motifs flanked by base-paired elements. Mutation of four guanosine residues in these two motifs - only 4 nucleotides per genomic RNA - reduced packaging to non-specific levels. These motifs only form in genomic RNA dimers; thus analysis of authentic full-length RNA was critical. This work illustrates how local context and RNA structure can create information-rich recognition signals from simple single-stranded sequence elements. In the second example, we discovered strong correlations between shRNA-mediated inhibition of HIV-1 replication in a cell-based assay and both the energetic cost of breaking secondary structures and the overall annealing energy between guide strand and folded target RNA. These correlations were only apparent when the energetic cost of disrupting pre-existing structure in the HIV-1 RNA was calculated using a secondary structure model derived from SHAPE probing information. We verified these correlations by design and evaluation of additional shRNAs and could achieve consistent inhibition of HIV-1 production by 90%. This approach emphasizes the profound, but ultimately predictable, role of RNA structure in tuning RNAi function and is likely to prove broadly useful in understanding large-scale biological recognition involving RNA.

